# Supplementary material for: Aberrant survival of hippocampal Cajal-Retzius cells leads to memory deficits, gamma rhythmopathies and susceptibility to seizures in adult mice
Source: Nat Commun. 2023 Mar 18;14:1531. doi: 10.1038/s41467-023-37249-7 (PMC10024761; doi:10.1038/s41467-023-37249-7)
Supplement: Supplementary file 1 — Supplementary Information [file 41467_2023_37249_MOESM1_ESM.pdf]

Aberrant survival of hippocampal Cajal-Retzius cells leads to memory deficits, gamma rhythmopathies and susceptibility to seizures in adulthood in mice

Martina Riva[1,2], Stéphanie Moriceau[3], Annunziato Morabito[4#], Elena Dossi[5#], Candela Sanchez-Bellot[6#], Patrick Azzam[1,2#], Andrea Navas-Olive[6], Beatriz Gal[6,7], Francesco Dori[1,2], Elena Cid[6], Fanny Ledonne[2], Sabrina David[8], Fabrice Trovero[8], Magali Bartolomucci[9], Eva Coppola[2], Nelson Rebola[4], Antoine Depaulis[9], Nathalie Rouach[5], Liset Menendez de la Prida[6], Franck Oury[3] and Alessandra Pierani [1,2,10*]

Supplementary Tables

Tested parameters		Males		Females	
		**Control** (N=17)	**Mutant** (N=8)	**Control** (N=9)	**Mutant** (N=9)
**Morphology**	Fur color	3.8 ± 0.2	4.0 ± 0.0	NA	NA
	Body weight after the test	20.5 ± 0.5	20.2 ± 0.6	18.8 ± 0.8	17.7 ± 0.6
	Body size	149.4 ± 1.4	149.1 ± 1.9	148.8 ± 2.7	148.1 ± 1.6
**Physical state**	Coat	0.0 ± 0.0	0.0 ± 0.0	0.0 ± 0.0	0.0 ± 0.0
	Groomed fur	0.0 ± 0.0	0.0 ± 0.0	0.0 ± 0.0	0.0 ± 0.0
	Marks of biting	0.0 ± 0.0	0.0 ± 0.0	0.0 ± 0.0	0.0 ± 0.0
	Body	0.0 ± 0.0	0.0 ± 0.0	0.0 ± 0.0	0.0 ± 0.0
	Whiskers	0.0 ± 0.0	0.0 ± 0.0	0.0 ± 0.0	0.0 ± 0.0
	Body tone	1.0 ± 0.0	1.0 ± 0.0	1.0 ± 0.0	1.0 ± 0.0
**Autonomic nervous system**	Respiration rate at the beginning of testing	2.0 ± 0.0	2.0 ± 0.0	2.0 ± 0.0	2.0 ± 0.0
	Respiration rate during testing	2.0 ± 0.0	1.8 ± 0.3	2.0 ± 0.0	2.0 ± 0.0
	Rectal temperature before testing	36.7 ± 0.2	36.7 ± 0.2	37.3 ± 0.4	37.2 ± 0.3
	Rectal temperature at the end of testing	36.9 ± 0.1	37.0 ± 0.1	37.5 ± 0.1	37.5 ± 0.2
	Salivation	0.0 ± 0.0	0.0 ± 0.0	0.0 ± 0.0	0.0 ± 0.0
	Pupil size	1.1 ± 0.0	1.1 ± 0.0	1.1 ± 0.1	1.0 ± 0.1
	Light-Pupil retraction	2.2 ± 0.1	2.5 ± 0.2	2.0± 0.0	2.0± 0.0
**Pain sensitivity**	Tail pinch	1.8 ± 0.2	1.9 ± 0.3	1.9 ± 0.1	1.7 ± 0.2
	Toe pinch	1.4 ± 0.3	1.5 ± 0.4	1.3 ± 0.2	1.7 ± 0.2
**Touch**	Pinna touching	2.0 ± 0.0	2.0 ± 0.0	2.1 ± 0.1	2.0 ± 0.0
	Vibrissae touching	2.1 ± 0.1	2.4 ± 0.2	2.4 ± 0.2	2.3 ± 0.2
	Finger approach - Finger slowly approched and held at 1 cm in front of the animal for 5s	2.0 ± 0.0	2.0 ± 0.0	NA	NA
	Head touch - Finger slowly approached until touching the head	2.0 ± 0.0	2.0 ± 0.0	2.0 ± 0.0	2.0 ± 0.0
	Touch escape (finger stroke from above)	1.5 ± 0.2	0.9 ± 0.2	1.6 ± 0.3	1.7 ± 0.2
**Vision**	Touch escape (finger stroke from above)	1.5 ± 0.2	0.9 ± 0.2	0.0 ± 0.0	0.0 ± 0.0
	Visual placing: hindpaws extension	2.1 ± 0.1	2.1 ± 0.1	0.0 ± 0.0	0.0 ± 0.0
**Audition**	Auditory response 'clic'	2.9 ± 0.1	3.1 ± 0.1	1.0 ± 0.0	1.0 ± 0.0
	Auditory response 'clap'	2.9 ± 0.1	2.6 ± 0.2	1.0 ± 0.0	1.0 ± 0.0
**Motricity Gait**	Gait	0.0 ± 0.0	0.0 ± 0.0	0.0 ± 0.0	0.0 ± 0.0
	Pelvic elevation during forward motion	2.0 ± 0.0	2.0 ± 0.0	2.0 ± 0.0	2.0 ± 0.0
	Tail elevation during forward motion	1.5 ± 0.1	1.4 ± 0.2	1.1 ± 0.1	1.2 ± 0.1
**Motricity Tonus Strength**	Grip strength	67.2 ± 3.9	64.2 ± 3.9	67.3 ± 3.6	75.3 ± 4.7
	Negative geotaxis	4.0 ± 0.0	4.0 ± 0.0	1.0 ± 0.0	1.0 ± 0.0
	Wire manoeuvre	0.5 ± 0.26	0.9 ± 0.4	0.6 ± 0.24	0.0 ± 0.0
	Hindlimb tone	2.0 ± 0.0	2.0 ± 0.0	2.0 ± 0.0	2.0 ± 0.0

Motricity Equilibrium	Fall from platform		0.1 ± 0.1	0.0 ± 0.0	0.0 ± 0.0	0.1 ± 0.1
	Righting reflex: time		0.0 ± 0.0	0.0 ± 0.0	0.0 ± 0.0	0.0 ± 0.0
	Negative geotaxis		6.5 ± 1.1	4.3 ± 1.1	7.9 ± 2.4	6.5 ± 2.8
	Equilibrium		0.0 ± 0.0	0.0 ± 0.0	0.0 ± 0.0	0.0 ± 0.0
	Wire maneuver		0.5 ± 0.3	0.9 ± 0.4	NA	NA
SNC tremors	Tremors		0.0 ± 0.0	0.0 ± 0.0	0.0 ± 0.0	0.0 ± 0.0
	Tremors		0.0 ± 0.0	0.0 ± 0.0	0.0 ± 0.0	0.0 ± 0.0
Reflex	Cornea		2.0 ± 0.0	2.0 ± 0.0	2.0 ± 0.0	2.0 ± 0.0
	Hind limb reflex		NA	NA	1.0 ± 0.0	1.0 ± 0.0
	Provoked biting		2.0 ± 0.0	2.0 ± 0.0	2.0 ± 0.0	2.0 ± 0.0
	Hindlimb tone		2.0 ± 0.0	2.0 ± 0.0	2.0 ± 0.0	2.0 ± 0.0
Reactivity	Pinna touching		2.0 ± 0.0	2.0 ± 0.0	2.1 ± 0.1	2.0 ± 0.0
	Vibrissae touching		2.1 ± 0.1	2.4 ± 0.2	2.4 ± 0.2	2.3 ± 0.2
	Finger approach - Finger slowly approched and held at 1 cm in front of the animal for 5s		2.0 ± 0.0	2.0 ± 0.0	2.0 ± 0.0	2.0 ± 0.0
	Head touch - Finger slowly approached until touching head		2.0 ± 0.0	2.0 ± 0.0	2.0 ± 0.0	2.0 ± 0.0
	Touch escape (finger stroke from above)		1.5 ± 0.2	0.9 ± 0.2	NA	NA
	Touch escape		1.1 ± 0.1	0.9 ± 0.2	1.2 ± 0.1	1.0 ± 0.2
	Resistance to manipulations		0.0 ± 0.0	0.0 ± 0.0	0.0 ± 0.0	0.0 ± 0.0
	Provoked biting		2.0 ± 0.0	2.0 ± 0.0	2.0 ± 0.0	2.0 ± 0.0
Activity in home cage	Body position		3.0 ± 0.0	3.0 ± 0.0	3.0 ± 0.0	3.0 ± 0.0
	Activity		1.9 ± 0.1	2.0 ± 0.0	2.0 ± 0.0	2.0 ± 0.0
	Awakening		3.8 ± 0.2	4.0 ± 0.0		
	Digging sawdust		0.2 ± 0.1	0.4 ± 0.2	0.4 ± 0.2	0.9 ± 0.2
	Grooming		0.4 ± 0.2	0.1 ± 0.1	0.6 ± 0.2	0.3 ± 0.2
Activity after transfer	Horiz A after transfer		63.3 ± 6.9	63.5 ± 7.0	80.7 ± 6.0	90.0 ± 8.8
	Vertic A after transfer		13.5 ± 1.8	16.3 ± 1.4	18.6 ± 2.4	17.1 ± 1.9
	Horizontal activity	From 0 to 30s	23.3 ± 1.5	25.8 ± 2.6	29.2 ± 2.1	31.6 ± 2.8
		From 30 to 120s	32.3 ± 3.5	40.1 ± 4.0	51.4 ± 4.1	58.4 ± 6.5
	Vertical activity	From 0 to 30s	3.5 ± 0.5	3.3 ± 0.9	4.2 ± 0.9	4.3 ± 0.8
		From 30 to 120s	7.9 ± 0.8	11.5 ± 1.8	14.3 ± 2.0	12.8 ± 1.4
	Palpebral closure		2.0 ± 0.0	2.0 ± 0.0	2.0 ± 0.0	2.0 ± 0.0
	Awakening		4.0 ± 0.0	4.0 ± 0.0	4.0 ± 0.0	4.0 ± 0.0
Activity after habituation	Palpebral closure		2.0 ± 0.0	2.0 ± 0.0	NA	NA
	Awakening		4.0 ± 0.0	4.0 ± 0.0	4.0 ± 0.0	4.0 ± 0.0
	Transfer on platform		2.0 ± 0.0	2.0 ± 0.0	2.0 ± 0.0	2.0 ± 0.0
	Descent latency		30.4 ± 0.6	31.0 ± 0.0	31.0 ± 0.0	28.9 ± 2.1
Freezing	Freezing		0.0 ± 0.0	0.0 ± 0.0	0.0 ± 0.0	0.0 ± 0.0
	Freezing after transfer		NA	NA	0.0 ± 0.0	0.0 ± 0.0
	Negative geotaxis		4.0 ± 0.0	4.0 ± 0.0	0.0 ± 0.0	0.0 ± 0.0
	Auditory response 'clic'		2.9 ± 0.1	3.1 ± 0.1	0.1 ± 0.1	0.0 ± 0.0
	Auditory response 'clap'		2.9 ± 0.1	2.6 ± 0.2	0.1 ± 0.1	0.0 ± 0.0
	Touch escape		1.1 ± 0.1	0.9 ± 0.2	0.2 ± 0.1	0.1 ± 0.1
Grooming	Grooming		0.4 ± 0.2	0.1 ± 0.1	0.6 ± 0.2	0.3 ± 0.2
	Groomings from 0 to 120s		0.5 ± 0.2	0.6 ± 0.2	0.2 ± 0.1	0.8 ± 0.2
Agressivity	Attack to touch		0.0 ± 0.0	0.0 ± 0.0	0.0 ± 0.0	0.1 ± 0.1
	Provoked biting		2.0 ± 0.0	2.0 ± 0.0	2.0 ± 0.0	2.0 ± 0.0

**Vocalisation**	Abnormal vocalisation	NA	NA	0.0 ± 0.0	0.0 ± 0.0
	Vocalisation to tail pinch	NA	NA	0.9 ± 0.1	0.7 ± 0.2
	Vocalisation to toe pinch	NA	NA	0.3 ± 0.2	0.7 ± 0.2
**Defecation**	Defecation	2.2 ± 0.1	2.0 ± 0.0	1.8 ± 0.1	1.9 ± 0.1
	Defecation	2.0 ± 0.1	2.0 ± 0.3	1.8 ± 0.1	1.8 ± 0.2
	Faeces consistency	2.0 ± 0.0	2.0 ± 0.0	2.0 ± 0.0	2.0 ± 0.0
**Urination**	Urination	1.0 ± 0.0	1.0 ± 0.0	0.4 ± 0.2	0.6 ± 0.2

Table S1: No changes in pain sensitivity, motricity gait, tonus strength and equilibrium in juvenile *BaxCKO* mutant mice. Primary screening of juvenile (28 to 35 days old) animals, males and females independently tested. The details of the tests and the scores used to evaluate mouse response are in Materials and Methods section. Comparisons between control and mutant animals were performed using the Mann-Whitney U test and no statistically significant differences were observed.

Figure	Experiment	N	Mean (Ctrl)	Mean (Mut)	SD (Ctrl)	SD (Mut)	p-value	test	Unit of mesurement	Age
**1A**	**Cookie test**	11 Ctrl, 11 Mut	232.3 (olfaction) ; 237.0 (motivation)	153.3 ; 157.4	175.5 ; 173.7	110.4 ; 110.0	p=0.2913 for olfaction; p= 0.2657 for motivation	Mann-Whitney	seconds	9 weeks (males)
**1A**	**Cookie test**	11 Ctrl, 11 Mut	248.8 (day 1) ; 124.0 (day2)	138.6 ; 67.4	173.9 ; 61.9	102.8 ; 58.6	F(1. 20)=4.466 p=0.0473 for genotype / p=0.027 for Ctrl. p=0.0539 for Mut	RM-two way ANOVA / Wilcoxon matched-pairs signed rank	seconds	9 weeks (males)
**1B**	**Y-maze**	14 Ctrl, 14 Mut	30.2 (number of entries) ; 56.51 (alternation)	32.5 ; 56.05	9.5 ; 9.1	11.5 ; 10.9	p=0.6912 for number of entries; p=0.9910 for percentage of alternation	Mann-Whitney	number of entries ; %	9 weeks (males)
**1C**	**Novel object recognition**	28 Ctrl, 23 Mut	0.562 (preference) ; 0.124 (discrimination)	0.478 ; -0.0438 ;	0.0493 ; 0.0985	0.0621 ; 0.124	p<0.0001 for preference and p<0.0001 for discrimination index	Mann-Whitney	%, %	Adults (males)
**1D**	**Novel object localization**	15 Ctrl, 19 Mut	0.586 (preference); 0.172 (discrimination)	0.474 ; -0.0502	0.0444 ; 0.0887	0.0369 ; 0.0739	p<0.0001 for preference and p<0.0001 for discrimination index	Mann-Whitney	%, %	Adults (males)
**1E**	**Morris Water Maze**	15 Ctrl, 19 Mut	20.64	39.28	SE of difference: 7.488		F(1. 31)=6.198 p=0.0184 for Genotype	RM-two way ANOVA	seconds	Adults (males)
**1F**	**Contextual Fear Conditioning**	18 Ctrl, 9 Mut	17.5 (training) ; 51.7 (testing)	24.2 ; 55.7	18.1 ; 14.8	11.4 ; 10.2	p=0.0757 for training; p=0.5612 for testing	Mann-Whitney	%	Adults (males)
**2A**	**CRs numbers (DAB staining)**	5 Ctrl, 5 Mut	84.34 (HF) ; 58.94 (vDG)	108.06 ; 62.75	6.06 ; 9.5	8.42 ; 11.85	p=0.0018 for HF; p=0.7709 for DG	2-way ANOVA, Sidak's multiple comparison	cell/mm	P24
**2B**	**CRs numbers (DAB staining)**	4 Ctrl, 4 Mut	54.32 (HF) ; 38.61 (vDG)	70.85 ; 39.29	2.41 ; 10.73	9.75 ; 8.52	p=0.0348 for HF; p=0.9922 for DG	2-way ANOVA, Sidak's multiple comparison	cell/mm	Adults
**2B**	**CRs numbers (IF P73)**	3 Ctrl, 3 Mut	51.34 (HF) ; 37.33 (vDG)	93.25 ; 37.71	7.76 ; 3.52	19.32 ; 12.10	p=0.0009 for HF; p=0.9984 for DG	2-way ANOVA, Sidak's multiple comparison	cell/mm	Adults
**2C**	**TOM-RELN-GABA colocalization**	4 Ctrl, 4 Mut	99.15% (TOM+/RELN+/GABA-) ; 0.85% (TOM+/RELN+/GABA+)	99.05% ; 0.95%	0.72 ; 0.72	0.80 ; 0.80	p<0.0001 for controls ; p<0.0001 for mutants	2-way ANOVA, Sidak's multiple comparison	%	Adults
**2C**	**CRs numbers (IF TOM)**	4 Ctrl, 4 Mut	58.19 (HF) ; 59.67 (vDG)	82.49 ; 46.09	7.76 ; 3.52	19.32 ; 12.10	p=0.0305 ; p=0.2615	2-way ANOVA, Sidak's multiple comparison	cell/mm	Adults
**3C**	**Power for theta and gamma bands in SLM**	17 Ctrl sessions, 16 Mut sessions (from 3 mice/group)	125.69 (theta); 107.66 (gamma)	123.36 (theta); 109.49 (gamma)	2.99 (theta); 2.61 (gamma)	2.64 (theta); 1.41 (gamma)	p=0.0211 (theta. top); p=0.04 (gamma. bottom)	Kruskal-Wallis test	dB	Adults
**3D**	**Power for theta and gamma bands in SP**	17 Ctrl sessions, 16 Mut sessions (from 3 mice/group)	116.75 (theta); 99.98 (gamma)	116.37 (theta); 103.05 (gamma)	2.92 (theta); 2.81 (gamma)	2.36 (theta); 1.94 (gamma)	p=0.614 (theta. top); p=0.0055 (gamma. bottom)	Kruskal-Wallis test	dB	Adults
**3E**	**Quantification of CR density along the CA1 HF**	13 Ctrl sections, 15 Mut sections (from 3 mice/group)	38.88	52.76	3.95	15.66	p=0.0321	Kruskal-Wallis test	cells/mm	Adults
**3F**	**Correlation between CR number and theta or gamma power**	For both theta and gamma: 13 sessions in Ctrl, 15 sessions in Mut (from 3 mice/group). Only sessions from which electrode track was recovered.	Gamma --- SLM: 107.9671; SP: 100.6403<br>Theta---- SLM: 125.9099 ; SP: 117.2661<br>CR density---- 38.88	Gamma --- SLM: 109.2713; SP: 102.9070<br>Theta---- SLM: 123.3406 ; SP: 116.3829<br>CR density---- 52.76	Gamma --- SLM: 2.3647; SP: 2.5501<br>Theta---- SLM: 2.9612 ; SP: 2.7687<br>CR density---- 3.95	Gamma --- SLM: 1.1304; SP: 1.9262<br>Theta---- SLM: 2.7307 ; SP: 2.4375<br>CR density---- 15.66	Gamma --- SLM: r=0.17. p=0.381; SP: r=0.39. p=0.04192<br>Theta---- SLM r=0.44. p=0.01933 ; SP: r=0.27. p=0.16933	Pearson correlation	CR density/dB	Adults
**4A**	**GAD67+ INs (ISH)**	3 Ctrl, 4 Mut	229.26 (CA1/CA2) ; 257.69 (CA3) ; 107.99 (DG)	202.63 ; 238.42 ; 95.89	42.54 ; 36.68 ; 7.71	51.58 ; 17.37 ; 28.36	p=0.6947 for CA1/CA2; p=0.8548 for DG; p=0.9578 for CA3	2-way ANOVA, Sidak's multiple comparison	cell/mm2	Adults
**4B**	**GAD67+ INs (ISH)**	4 Ctrl, 4 Mut	387.67 for CA1/CA2 ; 378.11 for CA3; 222.73 for DG	440.74 ; 378.17 ; 235.76	15.08 ; 22.62 ; 18.86	20.23 ; 27.26 ; 29.14	p=0.0118 for CA1/CA2; p=0.8129 for DG; p>0.9999 for CA3 (p=0.3429 for total)	2-way ANOVA, Sidak's multiple comparison (Mann-Whitney for total)	cell/mm2	P24
**4C**	**SST+ INs (ISH)**	3 Ctrl, 3 Mut	65.41 (CA1/CA2) ; 113.57 (CA3) ; 0 (DG)	69.43 ; 114.23 ; 0	7.91 ; 8.12 ; 0	5.78 ; 24.89 ; 0	p=0.9655 for CA1/CA2; p=0.9998 for DG; p>0.9999 for CA3 (p>0.9999 for total)	2-way ANOVA, Sidak's multiple comparison (Mann-Whitney for total)	cell/mm2	P24
**4D**	**NPY+ INs (IF)**	4 Ctrl, 4 Mut	29.89 (CA1/CA2) ; 24.15 (CA3) ; 5.8 (DG)	45.1 ; 26.34 ; 10.7	6.65 ; 2.97 ; 2.18	7.13 ; 3.41 ; 3.71	p=0.003 for CA1/CA2; p=0.5889 for DG; p=0.9621 for CA3 (p=0.0017 for total)	2-way ANOVA, Sidak's multiple comparison (Mann-Whitney for total)	cell/mm2	P24
**5A**	**Sholl Analysis (apical dendrites)**	8 Ctrl, 7 Mut	4.089	5.184	SE of difference: 0.4524		F(1. 13)= 5.862 p=0.0308 for genotype	RM-two way ANOVA	number of intersections	Adults
**5B**	**Number of spines**	6 Ctrl, 6 Mut (apical); 6 Ctrl, 7 Mut (basal)	1.03 (apical) ; 1.33 (basal)	1.59 ; 1.64	0.27 ; 0.237	0.344 ; 0.102	p=0.0260 for apical and 0.0350 for basal	Mann-Whitney	spines/µm	Adults
**5C**	**sEPSC**	11 Ctrl, 15 Mut	13.1 (amplitude) ; 1.72 (frequency)	13.2 ; 2.04	4.01 ; 0.742	3.12 ; 1.31	p=0.6461 amplitude; p=0.6832 frequency	Mann-Whitney	pA ; Hz	Adults
**5D**	**sIPSC**	15 Ctrl, 16 Mut	16.9 (amplitude) ; 4.91 (frequency)	15.2 ; 7.63	4.41 ; 2.95	3.77 ; 3.61	p=0.2995 amplitude; p=0.0405 frequency	Mann-Whitney	pA ; Hz	Adults
**6A**	**Adult seizures**	23 Ctrl, 16 Mut	26% (incidence) ; 4.74 (duration) ; 4.35% (mortality) ; 180 (latency)	94% ; 25.1 ; 62.5% ; 24.0	NA ; 10.1 ; 20.9 ; 103	NA ; 29.5 ; 50.0 ; 58.0	p<0.0001 for seizure incidence; p<0.0001 for duration; p=0.0001 for mortality and p<0.0001 for latency	Mann-Whitney	% ; minutes ; % ; minutes	Adults (males)
**6B**	**Duration**	23 Ctrl, 16 Mut	4.74	25.1	10.1	29.5	p<0.0001	Mann-Whitney	minutes	Adults (males)
**6C**	**Latency**	23 Ctrl, 16 Mut	180 ; 11.4 (responders only)	24 ; 9.64	103 ; 9.36	58 ; 7.38	p<0.0001 ; p=0.8348 (responders only)	Mann-Whitney	minutes	Adults (males)
**6D**	**Juvenile seizures**	7 Ctrl, 10 Mut	86% (incidence) ; 13.4 (duration) ; 71.4% (mortality) ; 24.6	100% ; 12.8 ; 70.0 % ; 26.3	0.976 ; 16.0 ; 48.8 ; 42.2	1.87 ; 10.0 ; 48.3 ; 34.3	p=0.4118 for seizure incidence; p=0.7611 for duration; p>0.9999 for mortality and p=0.5503 for latency	Mann-Whitney	% ; minutes ; % ; minutes	Juvenile (males)
**7C**	**type of activity**	20 Ctrl slices (4 mice), 20 Mut slices (4 mice)	bursting/seizures: 85%/15% (17/3 out of 20 slices)	bursting/seizures: 45%/55% (9/11 out of 20 slices)	N/A	N/A	p=0.0187	Fisher's exact test	%	Adults
**7D**	**seizure frequency and duration**	3 Ctrl slices, 11 Mut slices	0.29 seizures/min (frequency); 33.5 s (duration)	0.41 (frequency); 29.1 (duration)	0.08; 5.238	0.24; 20.04	no test because only n=3 for ctrl slices	N/A	seizures/minutes	Adults
**7E**	**seizure propagation**	11 Ctrl seizures, 37 Mut seizures	DG ; CA3 ; CA1 ;Sub; Ent; PRh/Ect: 33. 67. 100. 100. 100. 100 %	DG ; CA3 ; CA1 ; Sub.; Ent; PRh/Ect: 80. 100. 100. 100. 100. 100 %	N/A	N/A	p=0.0011	Chi-square test	seconds	Adults
**7F**	**seizure initiation sites**	11 Ctrl seizures, 37 Mut seizures	DG ; CA3 ; CA1 ;Sub; Ent; PRh/Ect: 0. 27.2. 9.1. 18.2. 9.1. 36.4 %	DG ; CA3 ; CA1 ; Sub.; Ent; PRh/Ect: 24.4. 27. 0. 5.4. 18.9. 24.3 %	N/A	N/A	ctrl vs Mut: p=1.000 (cortex vs hippocampal formation); p=0.6065 (Ent vs PRh/Ect); p=0.035 (hippocampal subregions)	Fisher's exact test; Fisher's exact test; Chi-square test	%	Adults

**S1A**	**Open field**	12 Ctrl, 12 Mut	1604	1877	SE of difference: 185.5		F(1. 22)=2.164 p=0.1554 for genotype	RM-two way ANOVA	mm	6 weeks (males)
**S1B**	**Elevated plus maze (EPM)**	14 Ctrl, 14 Mut	20.87	15.32	13.74	11.79	p=0.2219	Mann-Whitney	seconds	6 weeks (males)
**S1C**	**3-chambers**	12 Ctrl, 12 Mut	140.2 (stranger) ; 91.2 (empty)	155.4 ; 85.8	41.1 ; 38.1	52.1 ; 32.9	p=0.0179 for controls; p=0.0010 for mutants	RM two-way ANOVA, Sidak's multiple comparison	seconds	8 weeks (males)
**S1D**	**Forced swim test**	11 Ctrl, 12 Mut	65.65 (swim) ; 22.09 (climb)	67.73 ; 28.50	16.39 ; 13.55	8.501 ; 21.65	p=0.347 for swim; p=0.5254 for climb	Mann-Whitney	seconds	7 weeks (males)
**S1E**	**Rotaroad**	12 Ctrl, 12 Mut	328.8	441.3	169.9	161.2	p=0.0848	Mann-Whitney	seconds	8 weeks (males)
**S1F**	**Open field**	13 Ctrl, 5 Mut	11.09 (% time in center) ; 23.22 (% distance in center) ; 89.87 (total distance)	8.858 ; 19.79 ; 89.69	6.201 ; 10.88 ; 19.48	3.196 ; 8.416 ; 18.31	p=0.7028 for time in center. p=0.7028 for distance in center. p=0.8490 for total distance	Mann-Whitney	% ; %, cm	Adult (males)
**S1G**	**Dark and light**	13 Ctrl, 5 Mut	34.3 (% time in light) ; 12.9 (number of entries) ; 34.2 (% distance in light)	25.1 ; 11.2 ; 26.9	16.6 ; 7.34 ; 14.7	16.4 ; 5.67 ; 16.2	p=0.1936 for % time in light; p=0.2274 for number of entries; p=0.1936 % for distance in light	Mann-Whitney	%, number of entries, %	Adult (males)
**S2A**	**Novel object recognition**	27 Ctrl, 34 Mut	0.57 (preference) ; 0.141 (discrimination)	0.492 ; -0.0168	0.0372 ; 0.0743	0.0812 ; 0.162	p<0.0001 for preference and p<0.0001 for discrimination index	Mann-Whitney	%, %	Adult (females)
**S2B**	**Novel object localization**	22 Ctrl, 23 Mut	0.56 (preference) ; 0.120 (discrimination)	0.472 ; -0.0562	0.0732 ; 0.146	0.0523 ; 0.105	p<0.0001 for preference and p<0.0001 for discrimination index	Mann-Whitney	%, %	Adult (females)
**S2C**	**Morris Water Maze**	21 Ctrl, 23 Mut	23.15	46.98	SE of difference: 6.481		F(1. 42)=13.52 p=0.0007 for genotype	RM-two way ANOVA	seconds	Adult (females)
**S2D**	**Contextual Fear Conditioning**	12 Ctrl, 18 Mut	11.7 (training) ; 38.4 (testing)	9.19 ; 40.2	13.9 ; 25.4	7.76 ; 23.8	p=0.8265 for training. p=0.2106 for testing	Mann-Whitney	%	Adult (females)
**S2E**	**Open field**	7 Ctrl, 13 Mut	12.4 (time in centrer) ; 25.9 (distance in center) ; 113 (total distance)	15.9 ; 28.3 ; 130	12.4 ; 5.19 ; 25.1	15.9 ; 11.0 ; 31.8	p=0.2749 for % time in center; p=0.7573 for % distance in center; p=0.3929 for total distance	Mann-Whitney	% ; %, cm	Adult (females)
**S2F**	**Dark and light**	7 Ctrl, 13 Mut	25.3 (number of entries) ; 38.8 (distance in light) ; 32.0 (time in light)	24 ; 42.1 ; 39.2	6.1 ; 7.82 ; 8.37	9.69 ; 15.3 ; 14.7	p=0.3857 for number of entries ; p=0.1207 for distance in light; p=0.1255 time in light	Mann-Whitney	number of entries, % , %	Adult (females)
**S3A**	**CRs numbers (DAB staining)**	3 Ctrl, 5 Mut	64.3	109	11.7	26.7	p=0.0357	Mann-Whitney	cell/mm	P24
**S3B**	**WB Reelin**	4 Ctrl, 4 Mut	0.133	0.127	0.0508	0.0211	p>0.9999	Mann-Whitney	Total RELN/α-tubulin	Adult
**S4A**	**SST Adult**	4 Ctrl, 4 Mut	42.11 (CA1/CA2) ; 46.01 (CA3) ; 12.58 (DG)	45.67 ; 51.83 ; 18.96	10.33 ; 7.54 ; 4.30	14.06 ; 4.71 ; 3.85	p=0.9113 for CA1/CA2 ; p=0.7086 for CA3 ; p= 0.6471 for DG	2-way ANOVA, Sidak's multiple comparison	cell/mm2	Adult
**S4B**	**NPY adult**	5 Ctrl, 5 Mut	67.89 (CA1/CA2) ; 42.31 (CA3) ; 26.99 (DG)	56.18 ; 42.94 ; 20.71	43.76 ; 24.14 ; 13.75	30.51 ; 13.42 ; 12.41	p=0.9527 for CA1/CA2 ; p>0.9999 for CA3 ; p= 0.8519 for DG	2-way ANOVA, Sidak's multiple comparison	cell/mm2	Adult
**S5A**	**Filament lenght**	8 Ctrl, 7Mut for apical ; 6 Ctrl, 4 Mut for basal	4210 (apical) ; 3034 (basal)	5194 ; 3308	878 ; 352	764 ; 1269	p=0.0289 for apical ; p=0.9143 for basal	Mann-Whitney	mm	Adult
**S5B**	**Number of branches**	6 Ctrl, 4 Mut	55.3 (apical) ; 34.0 (basal)	67.1 ; 37.5	15.9 ; 6.13	15.8 ; 8.58	p=0.244 for apical ; p=0.5333 for basal	Mann-Whitney	number of branches	Adult
**S5C**	**Sholl Analysis (basal dendrites)**	5 Ctrl, 4 Mut	9.157	9.893	SE of difference: 1.834		F(1. 7)=0.1609. p=0.7003 for genotype	RM-two way ANOVA	number of intersections	Adult
**S5D**	**mEPSCs**	16 Ctrl, 10 Mut	13.3 (amplitude) ; 1.21 (frequency)	11.4 ; 1.47	2.96 ; 0.612	2.32 ; 0.855	p=0.1246 for amplitude ; p=0.5955 for frequency	Mann-Whitney	pA ; Hz	Adult
**S5E**	**Firing frequency**	21 Ctrl, 21 Mut	13.23	10.61	SE of difference: 2.614		F(1.000. 20.00)=2.418. p=0.1356 for genotype	RM-two way ANOVA	Hz	Adult
**S5F**	**Resting membrane potential**	21 Ctrl, 21 Mut	-59.1	-59.5	6.84	5.34	p=0.9405	Mann-Whitney	mV	Adult
**S5G**	**Action potential threshold**	21 Ctrl, 21 Mut	-35.3	-35.8	5.57	3.42	p=0.6585	Mann-Whitney	mV	Adult
**S5H**	**Input resistance**	21 Ctrl, 21 Mut	109	91.9	55.9	33.8	p=0.2202	Mann-Whitney	MOhm	Adult
**S6A**	**Adult Seizures**	7 Ctrl, 7 Mut	29% (incidence) ; 0.714 (duration) ; 0 % (mortality) ; 90.4 (latency)	86% ; 23.0 ; 42.9 % ; 27.3	0.976 ; 1.25 ; 0.00 ; 50.6	1.35 ; 20.1 ; 53.5 ; 41.6	p=0.0306 for seizure incidence; p=0.0064 for duration; p=0.055 for mortality and p=0.0361 for latency	Mann-Whitney	% ; minutes ; % ; minutes	Adult (females)
**S6B**	**Juvenile Seizures**	5 Ctrl, 7 Mut	100% (incidence) ; 18.7 (duration) ; 60.0% (mortality) ; 13.4 (latency)	86% ; 14.8 ; 57.1 % ; 29.8	2.3 ; 13.2 ; 54.8 ; 7.44	2.23 ; 13.6 ; 53.5 ; 40.1	p=0.4241 for seizure incidence; p=0.7866 for duration; p>0.9999 for mortality and p=0.4571 for latency	Mann-Whitney	% ; minutes ; % ; minutes	Juvenile (females)

Table S2: Summary of experimental data, parameters, sex and statistical analysis.

Aberrant survival of hippocampal Cajal-Retzius cells leads to memory deficits, gamma rhythmopathies and susceptibility to seizures in adulthood in mice

Martina Riva[1,2], Stéphanie Moriceau[3], Annunziato Morabito[4#], Elena Dossi[5#], Candela Sanchez-Bellot[6#], Patrick Azzam[1,2#], Andrea Navas-Olive[6], Beatriz Gal[6,7], Francesco Dori[1,2], Elena Cid[6], Fanny Ledonne[2], Sabrina David[8], Fabrice Trovero[8], Magali Bartolomucci[9], Eva Coppola[2], Nelson Rebola[4], Antoine Depaulis[9], Nathalie Rouach[5], Liset Menendez de la Prida[6], Franck Oury[3] and Alessandra Pierani [1,2,10*]

Supplementary Figures

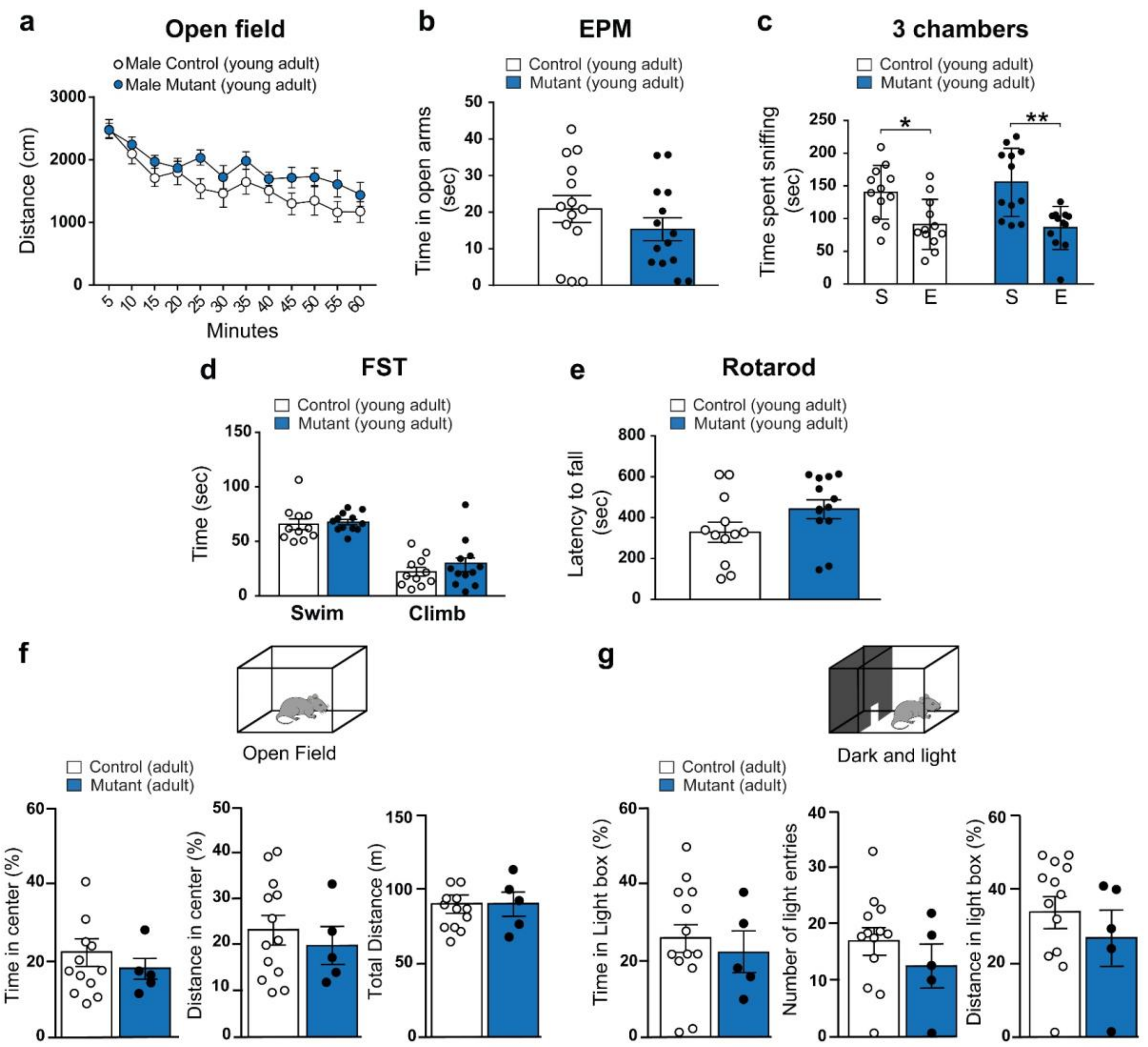
a
Open field
Male Control (young adult)
Male Mutant (young adult)
Distance (cm)
Minutes
b
EPM
Control (young adult)
Mutant (young adult)
Time in open arms (sec)
c
3 chambers
Control (young adult)
Mutant (young adult)
Time spent sniffing (sec)
S
E
d
FST
Control (young adult)
Mutant (young adult)
Time (sec)
Swim
Climb
e
Rotarod
Control (young adult)
Mutant (young adult)
Latency to fall (sec)
f
Open Field
Control (adult)
Mutant (adult)
Time in center (%)
Distance in center (%)
Total Distance (m)
g
Dark and light
Control (adult)
Mutant (adult)
Time in Light box (%)
Number of light entries
Distance in light box (%)

Figure S1

Fig. S1: *BaxCKO* mutant mice show normal locomotion, anxiety and sociability. a Open field test (OF) performed on young adults (6 weeks-old) male animals showing no differences in traveled distance between controls (n=12) and mutant (n=12) animals (F (1, 22) = 2.164; p=0.1554 RM Two-way ANOVA). **b** Elevated plus maze (EPM) performed on young adults (6 weeks-old) male animals showing no differences in time spent in the open arms (OA) between controls (n=14) and mutant (n=14) animals (p=0.2219 Mann-Whitney test). **c** 3-chamber sociability test performed on young adults (8 weeks-old) male animals. Both controls (n=12) and mutant (n=12) spent significantly more time sniffing the cage with a stranger (S) then the empty cage (E) (p=0.0179 for controls and p=0.0010 for mutants, RM Two-way ANOVA, post hoc Sidak's multiple comparison test). **d** Forced swim test (FST) performed on young adults (7 weeks-old) male animals showing no differences in the time spent swimming or climbing between controls (n=12) and mutant (n=12) animals (p=0.347 for swim, p=0.5254 for climb, Mann-Whitney test). **e** Rotarod test performed on young adults (8 weeks-old) male animals indicating no differences in latency to fall between controls (n=12) and mutant (n=12) animals (p=0.0848 Mann-Whitney test). **f** OF test performed in adult male (n=5 mutants and n=13 controls, Mann-Whitney test) animals. % of the time spent in the center versus the periphery (p=0.7028), % of the distance traveled in the center versus the periphery (p=0.7028) and the Total distance (m) (p=0.8490) were measured. No differences were detected between controls and mutants. **g** Light/Dark transition Test (L/DT) performed in adult males (n=5 mutants and n=13 controls, Mann-Whitney test). % time spent in the light compartment (p=0.1936), the number of entries in the light compartment (p=0.2274) and % of the distance traveled (p=0.1936) were measured. No differences were detected between controls and mutants. Asterisks indicate statistical significance (**, p<0.01). All data are presented as Mean ± SEM.

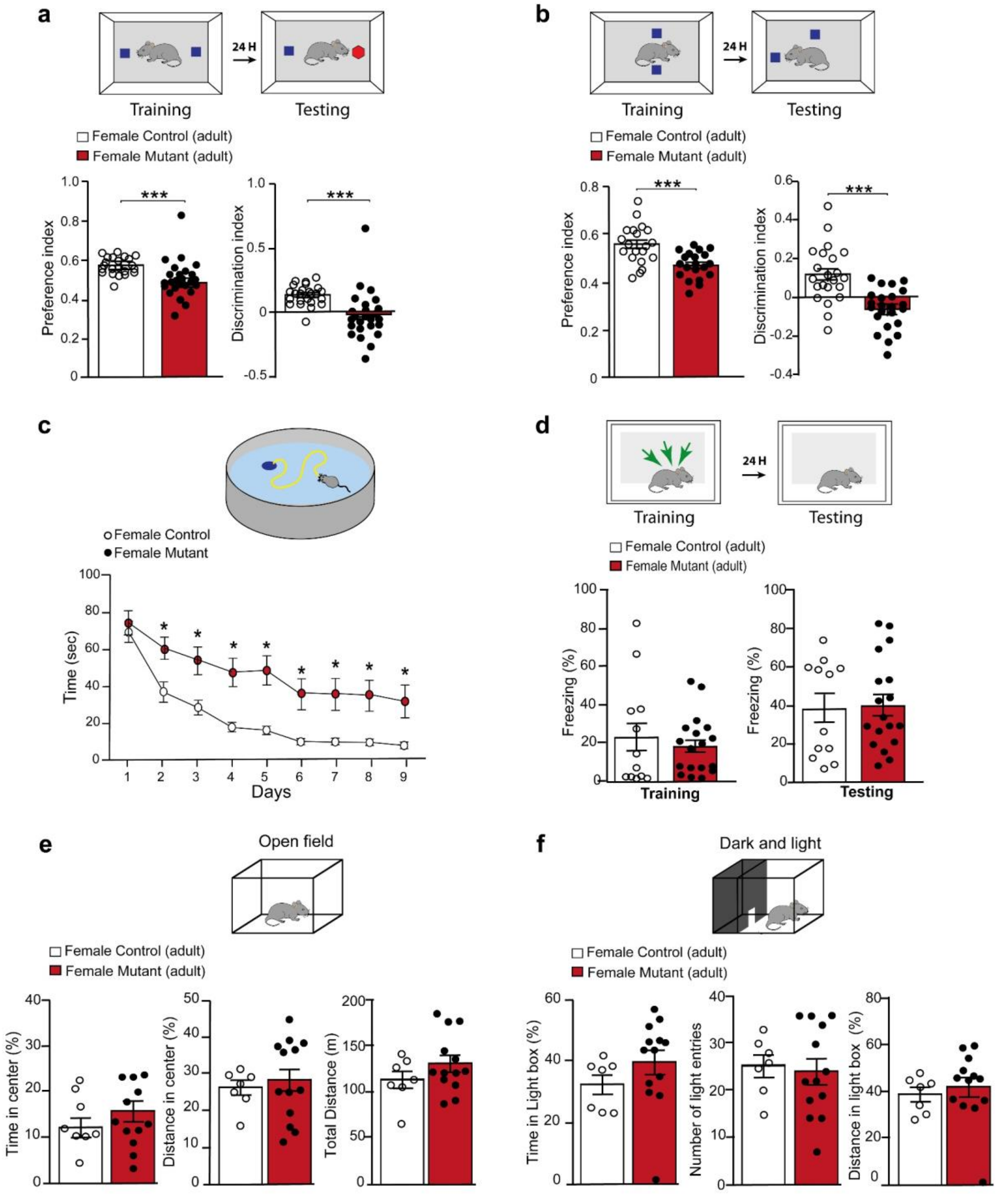

a
24 H
Training
Testing
Female Control (adult)
Female Mutant (adult)
Preference index
Discrimination index
***
b
Days
c
Female Control
Female Mutant
Time (sec)
d
Freezing (%)
Training
Testing
e
Open field
Time in center (%)
Distance in center (%)
Total Distance (m)
f
Dark and light
Time in Light box (%)
Number of light entries
Distance in light box (%)

Figure S2

Fig. S2: Adult *BaxCKO* mutant female mice exhibit deficits in hippocampal associated memory. **a** NOR test performed on three independent groups of adult female mutant animals and their control littermates (n=34) and controls (n=27). Preference and Discrimination index were measured 24 h after the training phase to assess memory performance. Mutant animals showed impairments in discriminating the new object (p<0.0001 and p<0.0001 for preference and discrimination index respectively, Mann-Whitney test). **b** NOL test performed on two independent groups of adult female mutant animals (n=22) and their controls littermates (n=23). Preference and Discrimination index were measured 24 h after the training phase. Mutant animals showed impairments in discriminating the new located object (p<0.0001 and p<0.0001 for preference and discrimination index respectively, Mann-Whitney test). **c** MWM test performed in adult female (n=23 mutants and n=21 controls) mice for 9 days. Mutant animals required more time to find the hidden platform (F (1, 42) = 13.52, p=0.0007 for Genotype, RM Two-way ANOVA). **d** Contextual Fear Conditioning (CFC) performed on females (mutant, n=18; control, n=12) from two independent groups. Percent freezing time was recorded during the training (as control for basal level, p=0.8265, Mann-Whitney test) and testing phases (to assess memory performance, p=0.2106, Mann-Whitney test). No differences were detected between control and mutant animals. **e** OF test performed in adult female mutant (n=13) and control (n=7) animals (Mann-Whitney test). % of the time spent in the center versus the periphery (p=0.2749), % of the distance traveled in the center versus the periphery (p=0.7573) and the Total distance (m) (p=0.3929) were measured. No differences were detected between controls and mutants. **f** Light/Dark transition Test (L/DT) performed in adult females (n=13 mutants and n=7 controls, Mann-Whitney test). % time spent in the light compartment (p=0.1255), number of entries in the light compartment (p=0.3857) and % of the distance traveled (p=0.1207) were measured. No differences were detected between controls and mutants. All tests were performed on 3 months-old female mice. Asterisks indicate statistical significance (*, p<0.05; ***, p<0.001). All data are presented as Mean ± SEM.

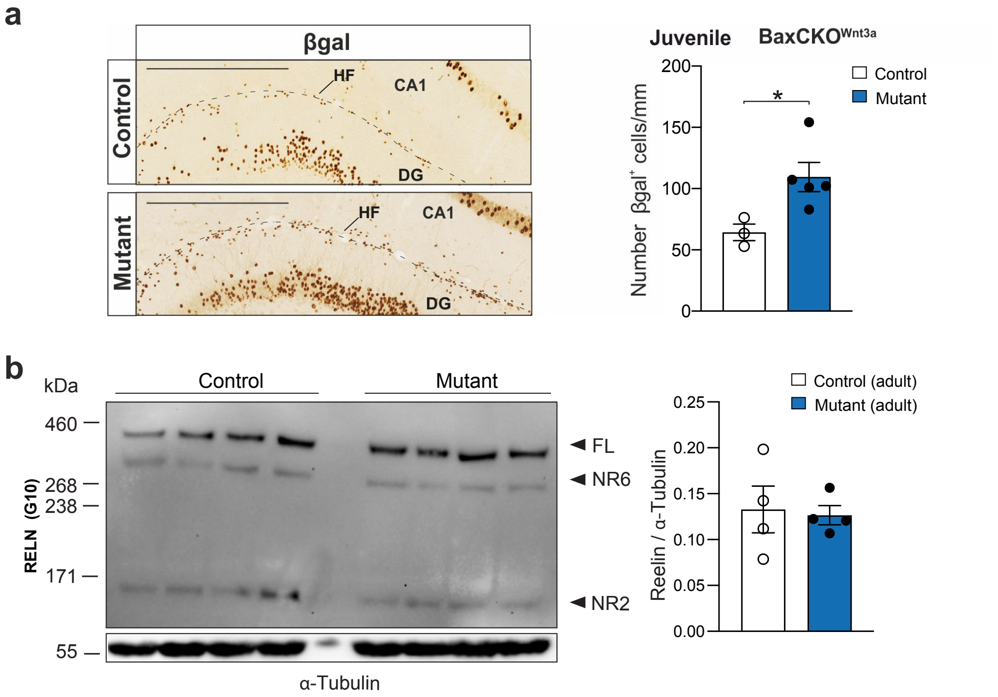

Figure S3

Fig. S3: Enhanced survival of CRs in the hippocampus of juvenile ***BaxCKO^{Wnt3aCre}*** **mutants. a** Coronal sections of control and *BaxCKO^{Wnt3acre}* (all the samples are carrying the *Tau^{nlsLacZ}* reporter) hippocampal region at P24 immunostained for βgal (left panels). Quantification of βgal^{+} cells in HF of control and mutant animals (right panels) showing increased number of CRs in mutants (controls: 64.25 ± 6.76 cells/mm; mutants: 109.5 ± 11.92 cells/mm, p=0.0357; Mann-Whitney test, n=3 controls and n=5 mutants). **b** Western blotting using an anti-Reln (G10) and anti-αtubulin (as internal control) showing no differences in the total amount of RELN protein in the hippocampus of adult controls and *BaxCKO* mice (p>0.9999; Mann-Whitney test, n=4 controls and n=4 mutants). All data are presented as Mean ± SEM.

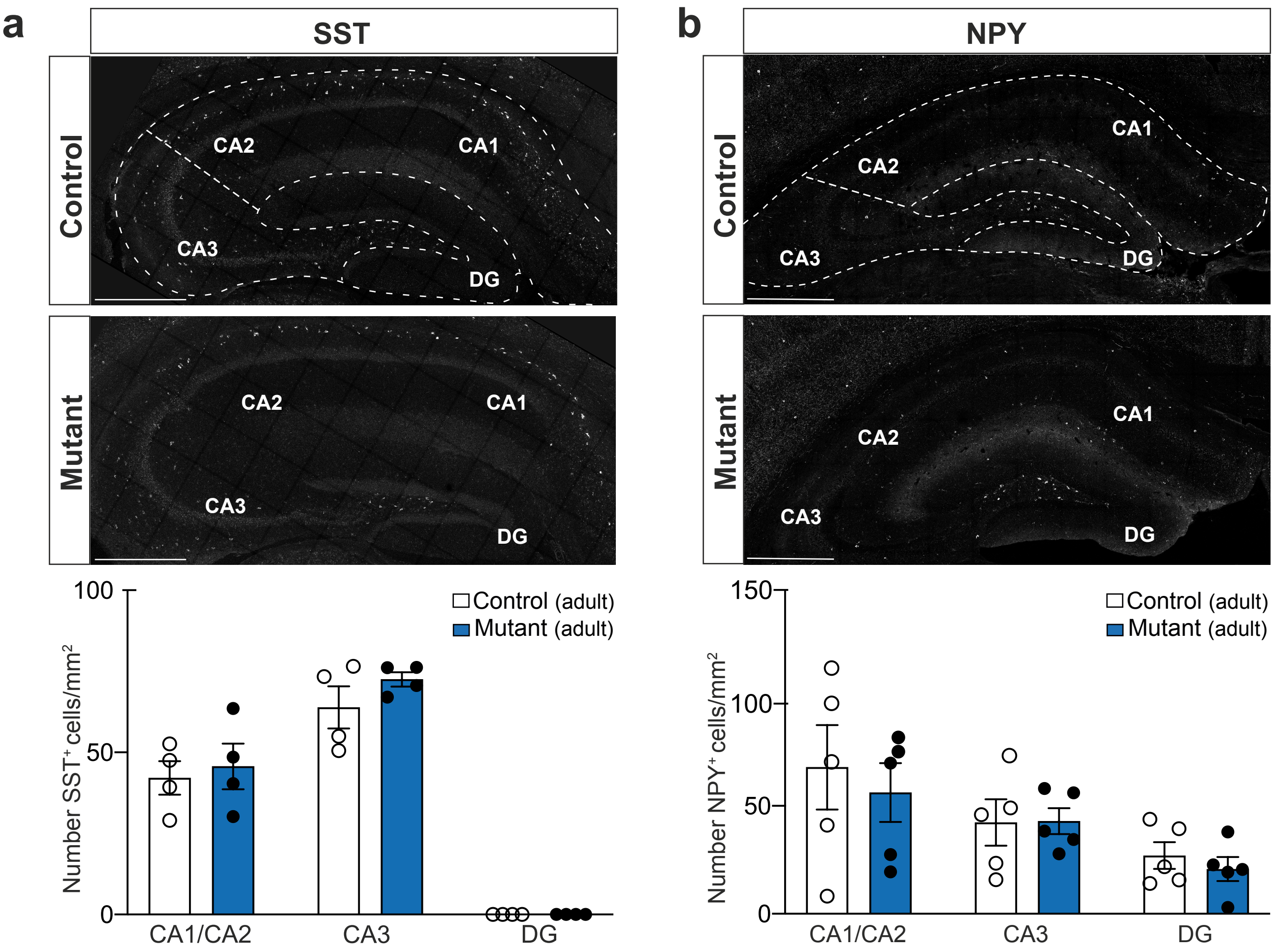
a
SST
Control
Mutant
CA1
CA2
CA3
DG
Number SST$^+$ cells/mm^2
Control (adult)
Mutant (adult)
100
50
0
CA1/CA2
CA3
DG
b
NPY
Control
Mutant
CA1
CA2
CA3
DG
Number NPY$^+$ cells/mm^2
Control (adult)
Mutant (adult)
150
100
50
0
CA1/CA2
CA3
DG

Figure S4

Fig. S4: Unaffected number of interneurons in the adult hippocampus. a Immunofluorescence of coronal sections from adult control and *BaxCKO* brains stained for SST (top panels). Quantification of SST^+ cells in the different hippocampal regions of control and mutant animals showing no differences (p=0.9291 for CA1/CA2; p=0.4788 for CA3; p>0.9999 for DG, two-way ANOVA, Sidak's multiple comparison test, n=4 controls and n=4 mutants). **b** Immunofluorescence of coronal sections from adult control and *BaxCKO* brains stained for NPY (top panels). Quantification of NPY^+ cells in the different hippocampal regions of control and mutant animals showing no differences (p=0.9527 for CA1/CA2; p>0.9999 for CA3; p=0.8519 for DG, two-way ANOVA, Sidak's multiple comparison test, n=5 controls and n=5 mutants). Dotted lines represent the subdivisions in different regions: CA1/CA2, CA3 and DG. Scale bar: 500 µm. All data are presented as Mean ± SEM.

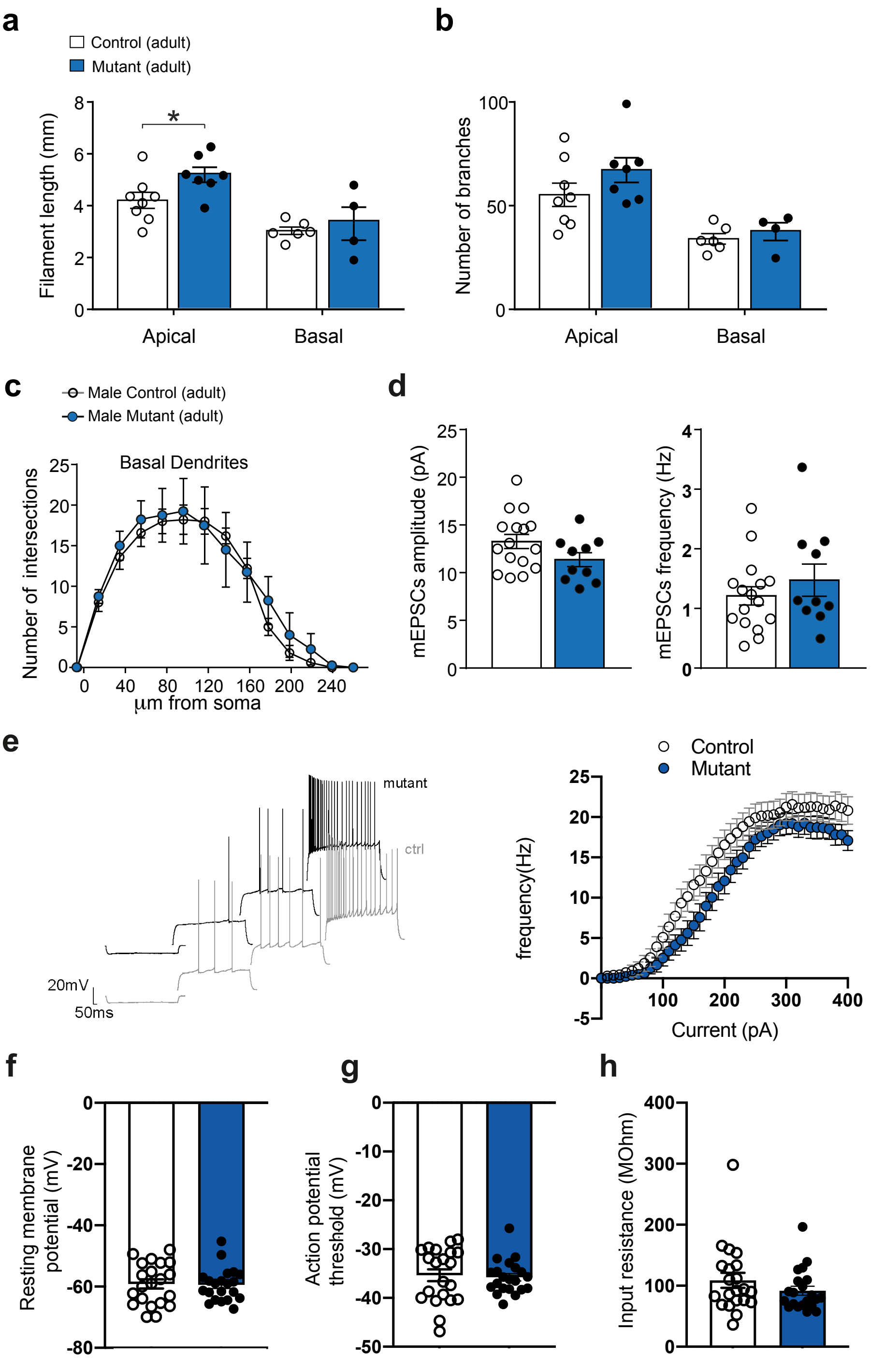
a
Control (adult)
Mutant (adult)
Filament length (mm)
Apical
Basal
b
Number of branches
Apical
Basal
c
Male Control (adult)
Male Mutant (adult)
Basal Dendrites
Number of intersections
µm from soma
d
mEPSCs amplitude (pA)
mEPSCs frequency (Hz)
e
mutant
ctrl
20mV
50ms
Control
Mutant
frequency(Hz)
Current (pA)
f
Resting membrane potential (mV)
g
Action potential threshold (mV)
h
Input resistance (MOhm)

Figure S5

Fig. S5: Analysis of CA1 pyramidal neurons morphology. a Increased filament length of apical (p=0.0289), but not basal (p=0.9143) dendrites in adult mutant animals (Mann-Whitney test, n=8 controls and 7 mutants for apical, n=6 controls and 4 mutants for basal dendrites). **b** No changes detected in the total number of branches (p=0.2440 for apical and 0.5333 for basal dendrites, Mann-Whitney test). **c** No changes detected in complexity (number of intersections) of basal dendrites (n=6 controls and 4 mutants, F(1, 7)=0.1609; p=0.7003 for genotype, RM Two-way ANOVA). **d** Summary plot of mEPSC amplitude and frequency estimated from individual CA1 pyramidal neurons from control and mutant mice. No statistically significant difference was observed between the two groups (p=0.1246 and 0.5955, respectively; n = 16 and 10 in controls and mutants, Mann-Whitey test). **e** *Left*: Representative current-clamp traces showing changes in the membrane potential of CA1 pyramidal neurons in response to somatic current injection steps of increasing amplitude (-90 pA,160 pA,170 pA and 290 pA) in both control and mutant mice. *Right*: Firing frequency as function of injected current showing no differences between control and mutant mice (F(1, 20)=2.418; p=0.1356 for genotype, RM two-way ANOVA). **f** Summary plot of resting membrane potential showing no differences between control and mutant mice (p=0.9405, Mann-Whitney test). **g** Same as F but for action potential threshold. Values were not statistically different between the two groups (p=0.6585, Mann-Whitney test). **h** Same as F but for input resistance (p=0.2202, Mann-Whitney test). Asterisks indicate statistical significance (*, p<0.05). All data are presented as Mean ± SEM.

a

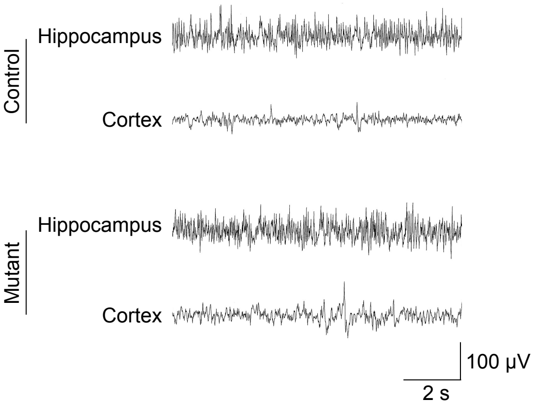

b

Adult females

Mouse Strain	Seizure incidence	Duration (min)	Mortality	Latency (min)
Control	2/7	0.71±0.47	0/7<br>0%	90.43±19.13
Mutant	6/7*	23.00±7.60 *	3/7<br>42.86%	27.29±15.74*

c

Juvenile females

Mouse Strain	Seizure incidence	Duration (min)	Mortality	Latency (min)
Control	5/5	18.65±5.92	3/5<br>60%	13.40±3.32
Mutant	6/7	14.79±5.13	4/7<br>57.14%	29.79±15.14

Figure S6

Fig. S6: Survival of CRs increases susceptibility to develop tonic-clonic seizures upon kainate injection in adult female mice. a Example of electroencephalographic recording in hippocampus and parietal cortex (Cortex) in control and mutant juvenile mice**. b** Results of kainate injection in adult female mice. Seizure incidence, duration, latency but not mortality, were increased in mutant females compared to littermate controls ($p=0.0306$, 0.0064, 0.0361 and 0.055 respectively; Mann Whitney test). **c** No change in any analyzed parameter was detected for juvenile females ($p=0.4241$ for seizure incidence; $p=0.7866$ for duration; $p>0.9999$ for mortality and $p=0.4571$ for latency, Mann Whitney test). Asterisks indicate statistical significance (*, $p<0.05$). Values are expressed as Mean ± SEM.
