# Supplementary material for: Aberrant survival of hippocampal Cajal-Retzius cells leads to memory deficits, gamma rhythmopathies and susceptibility to seizures in adult mice
Source: Nat Commun. 2023 Mar 18;14:1531. doi: 10.1038/s41467-023-37249-7 (PMC10024761; doi:10.1038/s41467-023-37249-7)
Supplement: Supplementary file 3 — Reporting Summary [file 41467_2023_37249_MOESM3_ESM.pdf]

nature portfolio

Corresponding author(s): Alessandra Pierani

Last updated by author(s): 22/02/23

Reporting Summary

Nature Portfolio wishes to improve the reproducibility of the work that we publish. This form provides structure for consistency and transparency in reporting. For further information on Nature Portfolio policies, see our Editorial Policies and the Editorial Policy Checklist.

Statistics

For all statistical analyses, confirm that the following items are present in the figure legend, table legend, main text, or Methods section.

n/a	Confirmed	
☐	☒	The exact sample size (*n*) for each experimental group/condition, given as a discrete number and unit of measurement
☐	☒	A statement on whether measurements were taken from distinct samples or whether the same sample was measured repeatedly
☐	☒	The statistical test(s) used AND whether they are one- or two-sided<br>*Only common tests should be described solely by name; describe more complex techniques in the Methods section.*
☒	☐	A description of all covariates tested
☐	☒	A description of any assumptions or corrections, such as tests of normality and adjustment for multiple comparisons
☐	☒	A full description of the statistical parameters including central tendency (e.g. means) or other basic estimates (e.g. regression coefficient) AND variation (e.g. standard deviation) or associated estimates of uncertainty (e.g. confidence intervals)
☐	☒	For null hypothesis testing, the test statistic (e.g. *F*, *t*, *r*) with confidence intervals, effect sizes, degrees of freedom and *P* value noted<br>*Give P values as exact values whenever suitable.*
☒	☐	For Bayesian analysis, information on the choice of priors and Markov chain Monte Carlo settings
☒	☐	For hierarchical and complex designs, identification of the appropriate level for tests and full reporting of outcomes
☐	☒	Estimates of effect sizes (e.g. Cohen's *d*, Pearson's *r*), indicating how they were calculated

Our web collection on statistics for biologists contains articles on many of the points above.

Software and code

Policy information about availability of computer code

Data collection

Images of histology were acquired with 20/40/63x objectives using confocal microscopes LEICA SP5/8 and/or with a slide scanner Nanozoomer 2.0 (20x) Hamamatsu). Data acquisition for Patch-clamp recordings was performed with Igor Pro 9 software and Neuromatic v3.0 extension. All recordings were low-pass filtered at 10 kHz and digitized at 100 kHz using an analog-to-digital converter (model NI USB 6259, National Instruments, Austin, TX, USA) and acquired using Neuromatic software (Rothman and Silver, 2018) running in Igor PRO (Wavemetrics, Lake Oswego, OR, USA). MEA recordings were collected with a Multichannel Systems (Germany), video microscope table (MEA-VMT1; Multichannel Systems, Germany) and MEA2100-120 system (bandwidth 1-3000 Hz, gain 5x, Multichannel Systems, Germany). In vivo recordings were collected using 16-channel silicon probes from Neuronexus, pre-amplification of LFP signals recorded at individual channels with multichannel amplifiers (Multichannel Systems) and digitized using a Digidata 1440A, Molecular Devices. Video-EEG activity was recorded with a digital acquisition computer-based system (Coherence, Deltamed, France). Data collection for behavioural tests used the Packwin 2.0 software (Bioseb (France), https://www.panlab.com/en/products/packwin-software-panlab) (for CFC), ActiMot2 Software (PhenoMaster Software, TSE System (Germany), https://www.tse-systems.com/productdetails/phenomaster/actimot?open=1228)/Acti-track (LSI Leticca, Panlab) (for OF/DL), Videotrack v2.6 Automated Behavioural Analysis (ViewPoint) tracking software (for EPM/3 chambers), CaptureStar v1 software (kainate injections). Blots were developed with SuperSignal West Pico Chemiluminescent Substrate and visualized on the ChemiDoc apparatus (Bio-Rad).

Data analysis

Softwares used for analysis: LAS AF software v2.6.0 build 7266 (Leica), Image J v1.53k (Schneider et al., 2012, https://imagej.nih.gov/ij/) and Fiji (NIH Image) for images/histology, IMARIS software 8.4 for 3D reconstruction and Graph Prism (v5) (GraphPad Software Inc., La Jolla, https:// www.graphpad.com/scientific-software/prism/) for statistical analysis; Neuromatic v3.0 software tools for Igor Pro 9 (Rothman and Silver, 2018) for Patch-clamp electrophysiology analysis; MEA Monitor v1.0.5 software and MC Rack 4.5.1 software (Multichannel Systems, Germany) and Neuroexplorer v4 (Nex Technologies, USA) for MEA experiments; Matlab 2021a (MathWorks) for Spectral analysis of LFP

activity. Video-EEG activity was analyzed with a digital acquisition computer-based system (Coherence, Deltamed, France). Quantitative blots' analysis was determined by densitometry using Image Lab™ v3.0 software (Bio-Rad).

For manuscripts utilizing custom algorithms or software that are central to the research but not yet described in published literature, software must be made available to editors and reviewers. We strongly encourage code deposition in a community repository (e.g. GitHub). See the Nature Portfolio guidelines for submitting code & software for further information.

Data

Policy information about availability of data

All manuscripts must include a data availability statement. This statement should provide the following information, where applicable:

- Accession codes, unique identifiers, or web links for publicly available datasets
- A description of any restrictions on data availability
- For clinical datasets or third party data, please ensure that the statement adheres to our policy

Source data are provided with this paper. All data are available in the main text or the supplementary materials.

Human research participants

Policy information about studies involving human research participants and Sex and Gender in Research.

Reporting on sex and gender	not applicable
Population characteristics	not applicable
Recruitment	not applicable
Ethics oversight	not applicable

Note that full information on the approval of the study protocol must also be provided in the manuscript.

Field-specific reporting

Please select the one below that is the best fit for your research. If you are not sure, read the appropriate sections before making your selection.

☒ Life sciences ☐ Behavioural & social sciences ☐ Ecological, evolutionary & environmental sciences

For a reference copy of the document with all sections, see nature.com/documents/nr-reporting-summary-flat.pdf

Life sciences study design

All studies must disclose on these points even when the disclosure is negative.

Sample size	No statistical method was used to predetermine sample sizes. The sample sizes and statistics used in this study are described in each figure legend. These are consistent with those of comparable previous anatomical (doi: 10.1016/j.celrep.2016.11.074; 10.7554/eLife.50503; 10.1038/s41467-021-26275-y; 10.1038/s41467-019-11904-4), behavioral (doi: 10.1016/j.cub.2018.12.021; 10.1084/jem.20201879) and functional studies (doi: 10.1371/journal.pbio.3001891; 10.1016/j.neuron.2022.05.016; 10.1523/JNEUROSCI.4297-14.2015;n). Sample sizes were chosen to support meaningful conclusions in accordance with ethical committee requirements to limit the use of animals, while being adequate in magnitude, to ensure statistical power to allow correct determination of statistical significance of results.
Data exclusions	Patch-clamp recordings: all recordings were not corrected for liquid junction potential and for all experiments data were discarded if series resistance, measured with a -5 mV pulse in voltage clamp configuration, was >20 MegaOhM or changed by more than 20% across the course of an experiment. No other data exclusions were made.
Replication	All experimental findings were collected on several animals from at least 3 different litters and confirmed the results. The exact number of replications for each experiment is detailed in each figure legend and statistical analysis/significance in text and figure legends and summarized in Table S2.
Randomization	For all the reported experiments randomization does not apply as there were no group allocation prior to the experiment. Animals were randomly assigned to experimental series tested the same day. We collected qualitative and quantitative assessments of the data across multiple individuals for each genotype (mutant and control) in each experimental series. In our experiments, group allocation corresponding to genotype was determined after data collection and analysis. Group allocation was determined by genotyping and sex for behavioural tests and genotyping for all others. Exclusively in experimental series for behavioural tests males and females were separated.
Blinding	The investigators were blinded to group allocation (control animals and mutants) during data collection and analysis.

Reporting for specific materials, systems and methods

We require information from authors about some types of materials, experimental systems and methods used in many studies. Here, indicate whether each material, system or method listed is relevant to your study. If you are not sure if a list item applies to your research, read the appropriate section before selecting a response.

Materials & experimental systems

n/a	Involved in the study
☐	☒ Antibodies
☒	☐ Eukaryotic cell lines
☒	☐ Palaeontology and archaeology
☐	☒ Animals and other organisms
☒	☐ Clinical data
☒	☐ Dual use research of concern

Methods

n/a	Involved in the study
☒	☐ ChIP-seq
☒	☐ Flow cytometry
☒	☐ MRI-based neuroimaging

Antibodies

Antibodies used

Primary antibodies: guinea pig anti-NPY (ab10341, Abcam, GR12360, 1:500), rabbit anti-GABA (A2052, Sigma, 045K4757, 1:2000), rat anti-SST (MAB354, Millipore, 3126964, 1:100), chicken anti-β-galactosidase (ab9361, Abcam, GR59431-2, 1:4000), goat anti-Reelin (AF3820, R&D Systems, YPE0320081, 1:500), mouse anti-Reelin (MAB5364, Millipore, 3494056, 1:2000), rabbit anti-alpha tubulin (PA529444, Invitrogen, WA3183690C, 1:1000) and rabbit anti-p73 (ab40658, Abcam, clone: EP436Y, G3416695-2, 1:500).

Secondary antibodies: donkey Cy3 anti-guinea pig (706-165-148, Jackson ImmunoResearch Laboratories, 151431, 1:700), donkey A488 anti-rabbit (711-545-152 Jackson ImmunoResearch Laboratories, 159922, 1:500), donkey Cy5 anti-rat (712-175-153 ImmunoResearch Laboratories, 121794, 1:250), donkey Cy5 anti-goat (705-175-147 ImmunoResearch Laboratories, 89840, 1:250), donkey A647 anti-rabbit (A32795, ThermoFisher, TJ271043, 1:200), biotinylated goat anti-chicken (103-065-155 Jackson ImmunoResearch Laboratories, 78100, 1:1000), HRP goat anti-mouse (115-035-008 Jackson ImmunoResearch Laboratories, 113193, 1:20000) and HRP Donkey anti-rabbit (111-035-008 Jackson ImmunoResearch Laboratories, 114506, 1:20000).

Validation

All antibodies used in this study were commercial and their validation is described by the manufacturers using either knock-out tissue, pre-immune serum before immunization and/or cell-type specific expression.

The p73 antibody ab40658 should recognize all p73 isoforms, including the delta-n isoforms, except for isoform 10 according to the uniprot file below: http://www.uniprot.org/uniprot/O15350. Suitable for: WB, Flow Cyt (Intra), ICC/IF, IHC-P, Unsuitable for: IP, Species reactivity Reacts with: Mouse, Human, Immunogen: Synthetic peptide. Positive control: WB: HeLa, Jurkat, HEK293 and NIH/3T3 cell lysates. IHC-P: Human urinary bladder carcinoma, human kidney, human liver carcinoma and mouse testis tissues. ICC/IF: HeLa cells. Refs: 1. Malik N et al. CBFB cooperates with p53 to maintain TAp73 expression and suppress breast cancer. PLoS Genet 17:e1009553 (2021). 2. Sadžak A et al. Neurotoxic Effect of Flavonol Myricetin in the Presence of Excess Copper. Molecules 26:N/A (2021).

The NPY antibody: species reactivity for mouse and rat as per the manufacturer, https://www.abcam.com/neuropeptide-y-antibody-ab10341.html Immunohistochemistry (PFA perfusion fixed frozen sections) - Anti-Neuropeptide Y antibody (ab10341) at a dilution of 1/500, staining Neuropeptide Y in rat hypothalamus by Immunohistochemistry (PFA perfusion fixed frozen sections). Refs: 1. Suzuki M et al. Anti-nerve growth factor therapy attenuates cutaneous hypersensitivity and musculoskeletal discomfort in mice with osteoporosis. Pain Rep 3:e652 (2018). 2. Yang JJ et al. KATP Channels Mediate Differential Metabolic Responses to Glucose Shortage of the Dorsomedial and Ventrolateral Oscillators in the Central Clock. Sci Rep 7:640 (2017). 3. Wu Q et al. Loss of GABAergic signaling by AgRP neurons to the parabrachial nucleus leads to starvation. Cell 137:1225-34 (2009).

The GABA antibody: species reactivity for mouse and rat as per the manufacturer, https://www.sigmaaldrich.com/ES/es/product/sigma/a2052. Expression of GABA in neocortical cells harvested from the brains of E19 day old rat embryos was detected by immunofluorescence using rabbit anti-GABA antibody. Triple IF staining was performed with the anti-GABA antibody and two anti-GAD antibodies. Expression of GABA was analyzed in cells isolated from the pallium of various animals including rats, mice, rabbits, guinea pigs, and lizards by immunohistochemistry. IHC was performed using rabbit anti-GABA antibody at 1:1000 diluted in a solution of 0.01M PBS ph 7.4 + 0.5% triton-x100. Rabbit anti-GABA antibody has been used for electron microscopy analysis in rat hippocampal tissues at a dilution of 1:4000 (R Fabian-Fine, P Skehel, M L Errington, H A Davies, E Sher, M G Stewart, A Fine. Ultrastructural distribution of the alpha7 nicotinic acetylcholine receptor subunit in rat hippocampus. J. Neurosci. (2001). The antibody has also been used for immunocytochemistry applications at dilutions ranging from 1:100-1:750 in Drosophila (R.I. Wilson, G. Laurent. Role of GABAergic inhibition in shaping odor-evoked spatiotemporal patterns in the Drosophila antennal lobe. J. Neurosci. (2005) and Periplaneta americana brain cells (A. Husch, M. Paehler, D. Fusca, L. Paeger, P. Kloppenburg. Calcium current diversity in physiologically different local interneuron types of the antennal lobe. J. Neurosci. (2009).

The SST antibody: species reactivity for mouse and rat as per the manufacturer, https://www.merckmillipore.com/ES/es/product/Anti-Somatostatin-Antibody-clone-YC7,MM_NF-MAB354#anchor_REF
Immunohistochemistry Analysis: Representative lot data. Formalin Fixed Paraffin Embedded (FFPE) rat brain tissue was processed using heat-induced epitope retrieval (HIER). Immunostaining was performed using a 1:100 dilution of Cat. No. MAB354, Anti-Somatostatin. Reactivity was detected using an HRP-conjugated anti-rat IgG secondary antibody and DAB. Positive staining was observed in interneurons of rat brain tissue. Refs: 1. Neurochemistry: Modern methods and applications. (1986). pp. 59-74. 2.

Immunological Reviews (1987). 100:279-306. 3. PNAS USA (1994). 91:2955-2959. 4. J. Neurosci (1996) 16:2701-2715. 5. J. Neurosci (1998) 18:1056-1071. 6. J. Neurosci. (1998) 18(17):6963-6976. 7. Pancreas (1999) 18:53-64.

The anti-β-galactosidase antibody: Immunohistochemistry (PFA perfusion fixed frozen sections) - Anti-beta Galactosidase antibody (ab9361). P0-adult mice were euthanized and perfused with 4% paraformaldehyde in PBS (PF). Their spinal cords were then post-fixed for 30–60 mins in 4% PF at 4°C (P0) or at room temperature (adult). Spinal cords were rinsed and cryoprotected in 20% sucrose in PBS (4°C) prior to embedding in OCT (Tissue-Tek). Immunostaining of frozen spinal sections was performed by incubating 20 µm thick sections with primary antibodies, which were then detected using species-specific secondary antibodies conjugated with Cy2, Cy3 and Cy5 or FITC. ab9361 was used at 1:1000. Immunohistochemistry (Formalin/PFA-fixed paraffin-embedded sections) - Anti-beta Galactosidase antibody (ab9361). ab9361 staining beta Galactosidase in mouse e13 stomach and liver tissue sections by Immunohistochemistry (IHC-P - paraformaldehyde-fixed, paraffin-embedded sections). Tissue was fixed with Davidson's fixative, permeabilized with 0.5% Triton-X 100 and blocked with 10% serum for 30 minutes at 22°C; antigen retrieval was by heat mediation in a citrate buffer. Samples were incubated with primary antibody (1/500 in TBST + 10% goat serum) for 16 hours at 4°C. A Biotin-conjugated goat anti-chicken IgY polyclonal (1/500) was used as the secondary antibody. Refs: 1. Marneros AG Magnesium and Calcium Homeostasis Depend on KCTD1 Function in the Distal Nephron. Cell Rep 34:108616 (2021). 2. Lowenstein ED et al. Olig3 regulates early cerebellar development. Elife 10:N/A (2021).

The RELN antibody: species reactivity for mouse as per the manufacturer, https://www.rndsystems.com/products/mouse-reelin-antibody_af3820. Reelin in Mouse Embryo. Reelin was detected in immersion fixed frozen sections of mouse embryo (13 d.p.c.) using Goat Anti-Mouse Reelin Antigen Affinity-purified Polyclonal Antibody (Catalog # AF3820) at 1.7 µg/mL overnight at 4 °C. Tissue was stained using the Anti-Goat HRP-DAB Cell & Tissue Staining Kit (brown; Catalog # CTS008) and counterstained with hematoxylin (blue). Specific staining was localized to developing brain. Refs: 1. S Venkataram, F Saaber, P Abe, D Schütz, PA Kumar, R Stumm. Cxcr4 and Ackr3 regulate allocation of caudal ganglionic eminence-derived interneurons to superficial cortical layers Cell Reports, 2022;40(5):111157. 2. A Rattner, CE Terrillion, C Jou, T Kleven, SF Hu, J Williams, Z Hou, M Aggarwal, S Mori, G Shin, LA Goff, MP Witter, M Pletnikov, AA Fenton, J Nathans. Developmental, cellular, and behavioral phenotypes in a mouse model of congenital hypoplasia of the dentate gyrus. Elife, 2020;9.

The RELN antibody: species reactivity for mouse as per the manufacturer, https://www.merckmillipore.com/FR/fr/product/Anti-Reelin-Antibody-a.a.-164-496-mreelin-clone-G10,MM_NF-MAB5364?ReferrerURL=https%3A%2F%2Fwww.google.com%2F. Detect Reelin using this Anti-Reelin Antibody, a.a. 164-496 mreelin, clone G10 validated for use in IH & WB. Mouse anti-Reelin (Catalog Number MAB5364) staining of embryonic cerebral cortex at 15 days in utero, with the pial surface above and the ventricle below. The positive zone is the Cajal-Retzius cell layer in the marginal zone, positive for reelin using MAB5364. Mouse anti-Reelin (Catalog Number MAB5364). Refs: 1. Sharaf, A; Rahhal, B; Spittau, B; Roussa, E. Localization of reelin signaling pathway components in murine midbrain and striatum. Cell and tissue research 359 393-407 (2015). 2. Trotter, JH; Lussier, AL; Psilos, KE; Mahoney, HL; Sponaugle, AE; Hoe, HS; Rebeck, GW; Weeber, EJ. Extracellular proteolysis of reelin by tissue plasminogen activator following synaptic potentiation. Neuroscience 274 299-307 (2014).

The alpha-tubuline antibody: TUBA1A Antibody (PA5-29444) in Western blot analysis was performed on whole cell extracts (30 µg lysate) of A549 (Lane 1), COS-7 (Lane 2), MDCK (Lane 3), C2C12 (Lane 4), MDA-MB-231 (Lane 5), PC-12 (Lane 6), RSC96 (Lane 7), tissue extracts of Mouse Lung (Lane 8) and Rat Brain (Lane 9). The blot was probed with Anti-alpha Tubulin Rabbit Polyclonal Antibody (Product # PA5-29444, 1:4000 dilution) and detected by chemiluminescence using Goat anti-Rabbit IgG (H+L) Superclonal™ Secondary Antibody, HRP conjugate (Product # A27036, 0.25 µg/mL, 1:4000 dilution). A 52 kDa band corresponding to alpha Tubulin was observed across the cell lines and tissues tested. Known quantity of protein samples were electrophoresed using Novex® NuPAGE® 4-12 % Bis-Tris gel (Product # NP0322BOX), XCell SureLock™ Electrophoresis System (Product # EI0002) and Novex® Sharp Pre-Stained Protein Standard (Product # LC5800). Resolved proteins were then transferred onto a nitrocellulose membrane with iBlot® 2 Dry Blotting System (Product # IB21001). The membrane was probed with the relevant primary and secondary Antibody following blocking with 5 % skimmed milk. Chemiluminescent detection was performed using Pierce™ ECL Western Blotting Substrate (Product # 32106). Ref: Lukacs M,Gilley J,Zhu Y,Orsomando G,Angeletti C,Liu J,Yang X,Park J,Hopkin RJ,Coleman MP,Zhai RG,Stottmann RW. Severe biallelic loss-of-function mutations in nicotinamide mononucleotide adenylyltransferase 2 (NMNAT2) in two fetuses with fetal akinesia deformation sequence. Exp Neurol (2019) 320:112961.
Cy™3 AffiniPure Donkey Anti-Guinea Pig IgG (H+L) (min X Bov, Ck, Gt, Sy Hms, Hrs, Hu, Ms, Rb, Rat, Shp Sr Prot) as per the manufacturer: https://www.jacksonimmuno.com/catalog/products/706-165-148. Ref: Single-cell transcriptomic analysis reveals diversity within mammalian spinal motor neurons. Nature Communications 2023 by Liau, E. S., Jin, S., et al.
Alexa Fluor® 488 AffiniPure Donkey Anti-Rabbit IgG (H+L) (min X Bov, Ck, Gt, GP, Sy Hms, Hrs, Hu, Ms, Rat, Shp Sr Prot). As per the manufacturer: https://www.jacksonimmuno.com/catalog/products/711-545-152. Ref: Meiotic DNA exchanges in C. elegans are promoted by proximity to the synaptonemal complex. Life Science Alliance 2023 by Almanzar, D. E., Gordon, S. G., et al.
Cy™5 AffiniPure Donkey Anti-Rat IgG (H+L) (min X Bov, Ck, Gt, GP, Sy Hms, Hrs, Hu, Ms, Rb, Shp Sr Prot). As per the manufacturer: https://www.jacksonimmuno.com/catalog/products/712-175-153. Ref: Ptbp1 knockdown failed to induce astrocytes to neurons in vivo. Gene Therapy 2023 by Yang, G., Yan, Z., et al.

The anti-chicken (103-065-155 Jackson ImmunoResearch Laboratories: Biotin-SP (long spacer) AffiniPure Goat Anti-Chicken IgY (IgG) (H+L) (min X Bov, Gt, GP, Sy Hms, Hrs, Hu, Ms, Rb, Rat, Shp Sr Prot). As per the manufacturer: https://www.jacksonimmuno.com/catalog/products/103-065-155. Ref: Phosphorylation and Pin1 binding to the LIC1 subunit selectively regulate mitotic dynein functions. Journal of Cell Biology (2021) by Kumari, A., Kumar, C., et al.

Cy™5 AffiniPure Donkey Anti-Goat IgG (H+L) (min X Ck, GP, Sy Hms, Hrs, Hu, Ms, Rb, Rat Sr Prot). As per the manufacturer: https://www.jacksonimmuno.com/catalog/products/705-175-147. Ref: FAM21 is critical for TLR2/CLEC4E-mediated dendritic cell function against Candida albicans. Life Science Alliance 2023 by Kulkarni, R., Kasani, S. K., et al.

Peroxidase AffiniPure Goat Anti-Mouse IgG, Fcγ fragment specific. As per the manufacturer: https://www.jacksonimmuno.com/catalog/products/115-035-008. Ref: Roles for the RNA-Binding Protein Caper in Reproductive Output in Drosophila melanogaster. Journal Developmental Biology 2022 by Tixtha, E. J., Super, M. K., et al.

Peroxidase AffiniPure Goat Anti-Rabbit IgG, Fc fragment specific. As per the manufacturer: https://www.jacksonimmuno.com/catalog/products/111-035-008. Ref: The assembly of mammalian SWI/SNF chromatin remodeling complexes is regulated by lysine-methylation dependent proteolysis. Nature Communications 2022 by Guo, P., Hoang, N., et al.

Animals and other research organisms

Policy information about studies involving animals; ARRIVE guidelines recommended for reporting animal research, and Sex and Gender in Research

Laboratory animals	ΔNp73CreIRESGFP(ΔNp73Cre) (Tissir et al., 2009) and TauloxP-stop-loxP-MARCKSeGFP-IRES-nlslacZ (TauGFP) (Hippenmeyer et al., 2005) and ROSA26 loxP-stop-loxP-Tomato (R26Tom) (Madisen et al., 2010) transgenic mice were kept in a C57BL/6J background. The Baxtm2Sjk;Bak1tm1Thsn/J line (Takeuchi et al., 2005) harboring the floxed Bax and the Bak knock-out alleles was purchased from the Jackson laboratory as mixed B6;129. Experiments were performed in both males and females aged juvenile (3-5 postnatal weeks), young adult (6-9 postnatal weeks) and adult mice (>3 months-old). All animals were handled in strict accordance with good animal practice as defined by the national animal welfare bodies at a temperature of 20°C and humidiy of 50%. The ΔNp73Cre line was crossed with the Baxtm2Sjk;Bak1tm1Thsn/J line (Baxlox/lox) to inactivate Bax function in specific CR subtypes and with the TauGFP and R26Tom reporter lines to permanently label CR subtypes. For histological analysis ΔNp73Cre+/-;Baxlox/+ animals were used as controls, while for behavioural and electrophysiological analysis ΔNp73Cre+/+;Baxlox/lox were used as control littermates. ΔNp73Cre +/- ;Baxlox/lox animals (BaxCKO) were used as mutants all along the study.
Wild animals	The study did not use wild animals.
Reporting on sex	Experiments were performed in both males and females. For behavioural tests results on males and females were independently performed and reported.
Field-collected samples	The study did not involve flield-collected samples.
Ethics oversight	For experiments at Paris, all animals were handled in strict accordance with good animal practice as defined by the national animal welfare bodies, and all mouse work was approved by the French Ministry of Higher Education, Research and Innovation as well as the Animal Experimentation Ethical Committee of Paris Descartes University (CEEA-34, licence numbers: 18011-201801261202754 N° 2018121415089383 – V8 APAFiS # 23831). For in vivo experiments in Madrid, all protocols and procedures were performed according to the Spanish legislation (R.D. 1201/2005 and L.32/2007) and the European Communities Council Directive 2003 (2003/65/CE) for animal research. Experiments were approved by the Ethics Committee of the Instituto Cajal and the Spanish Research Council (PROEX162-19).

Note that full information on the approval of the study protocol must also be provided in the manuscript.
